# Cigarette smoke induces nuclear translocation of heme oxygenase 1 (HO-1) in prostate cancer cells: Nuclear HO-1 promotes vascular endothelial growth factor secretion

**DOI:** 10.3892/ijo.2013.1910

**Published:** 2013-04-17

**Authors:** GABRIEL BIRRANE, HUCHUN LI, SUPING YANG, SOUVENIR D. TACHADO, SEYHA SENG

**Affiliations:** 1 Divisions of Experimental Medicine, Department of Medicine, Beth Israel Deaconess Medical Center, Harvard Medical School, Boston, MA 02215, USA; 2 Molecular and Vascular Medicine, Department of Medicine, Beth Israel Deaconess Medical Center, Harvard Medical School, Boston, MA 02215, USA; 3 Pulmonary, Critical Care and Sleep Medicine, Department of Medicine, Beth Israel Deaconess Medical Center, Harvard Medical School, Boston, MA 02215, USA

**Keywords:** cigarette smoke, nuclear heme oxygenase 1, vascular endothelial growth factor, prostate cancer

## Abstract

Prostate cancer is the second leading cause of male-cancer related death in the United States. Despite a number of evidence-based studies which strongly suggest an association between cigarette smoking and prostate cancer, the underlying biological mechanism is largely unknown. Heme oxygenase 1 (HO-1) has been implicated in maintaining cellular homeostasis, but also in tumor angiogenesis. Nuclear HO-1 protein expression has been observed in various types of tumors including prostate cancer. These studies, however, were reported as clinical and pathological observations, and failed to investigate nuclear HO-1 at the molecular level in cancer. The present study explores the relationship between cigarette smoke and nuclear HO-1-modulated promotion of vascular endothelial growth factor (VEGF) secretion. We have demonstrated that cigarette smoke medium (SM)-induced HO-1 mRNA expression and upregulated HO-1 protein levels in the prostate cancer cell lines DU145 and PC3. We also observed that SM significantly induced nuclear expression of HO-1, and enhanced secretion of VEGF in cells. Nuclear-directed expression of HO-1 activated the transcriptional activity of VEGF and promoted VEGF secretion in prostate cancer cells. This study provides new insights into the molecular mechanism by which cigarette smoke-induced nuclear translocation of HO-1 promotes VEGF secretion in prostate cancer cells. Nuclear HO-1 may, therefore, constitute an attractive therapeutic target to inhibit angiogenesis and the progression of prostate cancer.

## Introduction

Prostate cancer is the second leading cause of male-malignancy-related death in the United States (1). A growing body of data indicates that the initiation and progression of prostate cancer is influenced by aging, genetic predisposition, environmental factors such as toxins, and lifestyle choices such as cigarette smoking. Cigarette smoking has been identified as the most preventable cause of cancer morbidity and mortality, yet, 20% of US adult males were reported to be cigarette smokers in 2010 (2).

The combustion product of cigarettes is an aerosol containing more than 3,500 chemical compounds, many of which have been shown to be carcinogens or mutagens. Smoke generated from burned cigarettes consists of a particulate solid phase (tar) and a gaseous phase containing volatile organic compounds, free radicals and other volatile and semi-volatile compounds (3,4). The water-soluble components of aqueous cigarette tar can produce the superoxide anion (O_2_^•−^) and subsequently hydrogen peroxide (H_2_O_2_) and the reactive hydroxyl radical (HO^•^), which can cause oxidative stress damage to membrane lipids, proteins and DNA (4), contributing to inflammation and cancer. Active and passive exposure to products of cigarette combustion promotes angiogenesis and malignancy of the prostate, lung, esophagus, bladder, pancreas and cervix (5). Cigarette smoking has also been linked to an elevated risk of advanced stage and high-grade prostate cancer, both of which are indicative of a poor prognosis (6). Although much is known about the epidemiology of cigarette smoking, the underlying cellular and molecular mechanisms responsible for its carcinogenic potential in prostate cancer remain unclear.

Heme oxygenase (HO) is a microsomal rate-limiting enzyme involved in the degredation of heme (7,8). The three mammalian isoforms of heme oxygenase, HO-1, HO-2 and HO-3, have distinct patterns of tissue-specific expression (9). HO-1, also known as heat shock protein 32 (HSP-32), is highly expressed in the spleen and liver, and at lower levels in several other mammalian tissues (10,11) has been implicated in maintaining cellular homeostasis, reducing oxidative stress damage, attenuating the inflammatory response, inhibiting apoptosis and regulating proliferation (12). Conversely, HO-1 is also recognized as an important proangiogenic mediator (13). HO-1 expression is elevated in various cancer cells (14–17) and tumors (18–20). Ectopic expression of HO-1 has been shown to increase VEGF secretion and enhance VEGF-mediated activities such as proliferation and migration, leading to improved formation and growth of capillary-like tubular structures (21,22) as well as tumor angiogenesis in a mouse model of pancreatic cancer (21–23). Endothelial cells deficient in HO-1 secrete less VEGF than their wild-type counterparts. Reducing HO-1 expression or inhibiting its enzymatic activity impairs vGPCR-enhanced survival and VEGF-A expression in endothelial cells (24), and inhibits VEGF expression in lung carcinoma (25,26).

A number of cohort studies suggest that cigarette smoking may be associated with prostate cancer (6), however, the molecular mechanism(s) linking it to prostate cancer remain elusive. Nuclear HO-1 protein expression has been observed in various tumors (27–29) including prostate cancer (19). These studies, however, were reported as clinical and pathological observations, and failed to investigate role of nuclear HO-1 expression molecularly in prostate cancer. The present study explored the relationship between cigarette smoke and nuclear expression of HO-1 and to investigate molecular mechanism(s) by which cigarette smoke-induced nuclear translocation of HO-1 promoted VEGF secretion in prostate cancer cells. The present study demonstrated cigarette smoke induced nuclear translocation of HO-1 in prostate cancer cells. Nuclear-directed expression of HO-1 increased transcriptional activity and secretion of VEGF in prostate cancer cells. The data revealed that cigarette smoke-mediated translocation of HO-1 was associated with increased VEGF secretion, and also suggested that exposure to first- and secondhand products of cigarette combustion were associated with prostate cancer via nuclear HO-1-modulated VEGF secretion.

## Materials and methods

### Reagents

Anti-4-hydroxy-2-nonenal (anti-4-HNE) antibody (cat. 24325) was purchased from Percipio Biosciences (Burlingame, CA). Anti-GAPDH (cat. sc-137179), anti-lamin B1 (cat. Sc-56144) and anti-GFP (cat. sc-8334) antibodies were purchased from Santa Cruz Biotechnology (Santa Cruz, CA). Anti-HO-1 antibody (cat. ADI-SPA-895-F) was purchased from Enzo Life Sciences (Ann Arbor, MI). Titanium One-Step RT-PCR kit (cat. 639503) was purchased from Clontech (Mountain View, CA). Human VEGF ELISA kit (cat. DVE00) was purchased from R&D Systems (Minneapolis, MN). NE-PER Nuclear and Cytoplasmic Extraction Reagents (cat. 78833) were from Thermo Scientific (Rockford, IL). CellTiter 96 Aqueous non-radioactive cell proliferation assay kit (cat. G5421) was purchased from Promega Corporation (Madison, WI).

### Cells and culture conditions

The prostate carcinoma cell line DU145 (cat. HTB-81) was obtained from the American Type Culture Collection (Manassas, VA) and cultured in Eagle’s minimum essential medium (Mediatech Inc., Manassas, VA) supplemented with 10% FBS. The prostate adenocarcinoma cell line PC3 (cat. CRL-1435) was also obtained from the American Type Culture Collection and cultured in DMEM F-12 50/50 medium (Mediatech Inc.), supplemented with 10% FBS. COS-7 and HEK293T cells were cultured in Dulbecco’s modified Eagle’s medium (Mediatech Inc.) containing 10% FBS. All cells were maintained at 37°C, in a 5% CO_2_ incubator.

### Cigarettes and the preparation of smoke medium

3R4F reference cigarettes were purchased from the Reference Cigarette Program, University of Kentucky (Lexington, KY). Federal Trade Commission Smoking analysis indicated that 3R4F cigarettes contained 11.0 mg/cigarette (mg/cig) total particulate matter (TPM), 9.4 mg/cig of tar, 0.73 mg/cig nicotine, and 12 mg/cig carbon monoxide. Smoke media (SM) was generated by collecting whole smoke from burning one pack (20 cigarettes) of reference cigarettes into 100 ml of cell culture media. SM was filtered and stored at −80°C for further use.

### Cell proliferation assay

The assay was performed using a CellTiter 96 Aqueous non-radioactive cell proliferation assay kit per manufacturer’s instruction. Briefly, 100 *μ*l of DU145 and PC3 (5×10^4^ cells) cell suspension were grown on 96-well plates incubated at 37°C and 5% CO_2_. After 24 h, the culture medium was either refreshed or replaced with serial dilutions of SM as indicated. The accumulation of formazan was measured spectroscopically by absorption at 490 nm.

### RNA isolation

Isolation of total RNA was performed using TRIzol Reagent (Life Technologies, Carlsbad, CA) according to the manufacturer’s instructions. Briefly, cells were seeded on 6-well plates and treated with SM or standard cell culture media. A total of 1.0 ml of TRIzol was added followed by 0.2 ml of chloroform after 15 min. Samples were vigorously inverted by hand for 15 sec, incubated at room temperature for 3 min and centrifuged at 12,000 × g for 15 min at 4°C. After centrifugation, 0.5 ml of isopropanol was added to the supernatant. After incubation at room temperature for 10 min, samples were centrifuged at 12,000 × g for 10 min at 4°C. The pellets were washed with 75% ethanol, dissolved in RNAse-free water and incubated for 10 min at 60°C. RNA concentration was measured and the samples were stored at −80°C.

### RT-PCR Analyses

RNA transcript levels were semi-quantified using the Titanium One-Step RT-PCR kit (Clontech) according to the manufacturer’s instructions using the forward 5′-GAGACGGCTTCAAGCTGGTGATG-3′ and reverse 5′-GTTGAGCAGGAACGCAGTCTTGG-3′ primers for HO-1, and the forward 5′-GAAGGTGAAGGTCGGAGTC-3′ and reverse 5′-GAAGATGGTGATGGGATTTC-3′ primers for GAPDH. The conditions were one cycle of 50°C for 1 h and 94°C for 5 min, followed by 25 cycles of 94°C for 30 sec, 68°C for 30 sec and 68°C for 60 sec with an extension of 68°C for 2 min. The RT-PCR products were visualized on 1% agarose gels stained with ethidium bromide and quantified by Scion Image Software.

### Immunoblot analysis

Cells were washed with PBS and lysed directly on ice with RIPA buffer. The lysates were transferred to a new tube, solubilized for 1 h at 4°C and clarified by centrifugation at 12,000 rpm for 20 sec at 4°C. Total cell extract protein concentration was determined by Bradford assay. Equal amounts of proteins were loaded and electrophoresed on SDS-PAGE gels, blotted onto PVDF membrane and incubated with anti-HO-1 and anti-GAPDH antibodies. Blots were washed 3 times and incubated with a HRP-conjugated IgG antibody. Protein expression was detected on X-ray films and quantified using Scion Image Software.

### Immunohistochemistry

Cell cultures were washed twice with PBS, fixed with 4% paraformaldehyde and treated with 0.5% Triton X-100 in PBS for 30 min. After 3 washes with PBS, cells were treated with PBS containing 10% goat serum for 2 h and incubated with anti-4HNE antibodies for 24 h at 4°C. After 3 washes with PBS, the cells were incubated with FITC- and/or TRITC-conjugated IgG antibodies for 1 h at room temperature. The cells were then washed, mounted on slides and imaged using a Zeiss LSM510 Meta confocal microscope.

### Generation of DNA constructs

pEGFP-HO-1 (HO-1 fused with the C-terminus to EGFP) was created by PCR amplification of HO-1 cDNA using the forward primer 5′-CAGC*GAATTC* ACCATGGAGCGTCCGCAACCCGACAGC-3′ containing an *Eco*RI restriction site and the reverse primer 5′-GAT *GGATCC*CGATGCGGCCGCCATGGCATAAAGCCCTAC-3′ containing a *Bam*HI site. The product was ligated into the pEGFP-N3 vector at the *Eco*RI and *Bam*HI sites. Similarly, pEGFP-HO-1/NLS (same as pEGFP-HO-1, but containing tandem nuclear localization signals between HO-1 and EGFP) was created by PCR amplification of the pNuc-HO-1 template (described below) using the same forward primer as above and the reverse primer 5′-CTG*GGATCC*CTACCTTTCTCTTCTTTTTTGGATCTACCTTTCTCTTC-3′ containing a *Bam*HI site. The product was inserted into pEGFP-N3 at the unique *Eco*RI and *Bam*HI restriction sites.

pFlag-HO-1 (HO-1 with an N-terminal FLAG-tag) was created by PCR amplification of the HO-1 cDNA using the primers 5′-C*AAGCTT*GAGCGTCCGCAACCCGACAGC-3′ and 5′-C*GGATCC*TCATTACATGGCATAAAGCCC-3′, and insertion into the pFlag-CMV4 vector at the *Hin*dIII and *Bam*HI sites. pNuc-HO-1 (HO-1 with tandem nuclear localization at the C-terminus) was generated by PCR amplification using the primers 5′-TA*GTCGAC*GAGCGTCCGCAACCC GAC-3′ and 5′-TA*GCGGCCGC*CATGGCATAAAGCCCT-3′ and insertion into the pCMV/myc/nuc vector at *Sal*I and *Not*I restriction sites. Expression in HEK293T cells was confirmed by immunoblot analysis using an anti-HO-1 antibody.

### Luciferase assay

HEK293T or COS7 cells were co-transfected with combinations of plasmids as indicated in the figure legends using Lipofectamine reagent (Life Technologies). Cell lysates were prepared and luciferase and β-galactosidase activities were quantified using a Luciferase assay kit (Promega) according to the manufacturer’s instructions. The effect of the transfected proteins on promoter transcriptional activity was assessed by measuring luciferase activity normalized to β-galactosidase activity.

### Statistics

Statistical significance was determined using the Student’s t-test and one-way ANOVA. Data represent the mean ± SD of independent experiments, with a p<0.05 considered statistically significant.

## Results

### Effect of cigarette smoke on growth of prostate cancer cells

**Figure 1:**
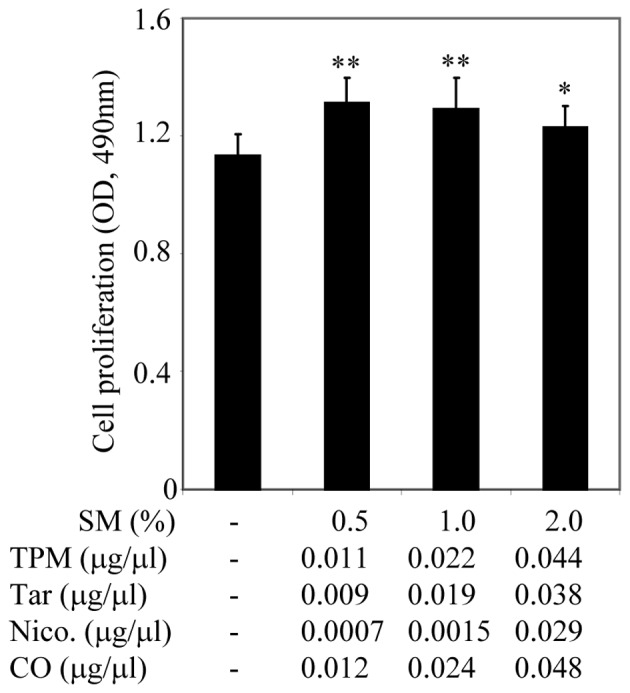
Effect of cigarette smoke on growth of prostate cancer cells. PC3 cells were plated onto 96-well plates and starved with culture medium containing 0.5% FBS. After 24 h, cell cultures were treated with serial dilutions of SM (2, 1 and 0.5%) as indicated. Concentrations of total particulate matter (TPM), tar, nicotine (Nico) and carbon monoxide (CO) were estimated based on information provided by the reference cigarette program. A CellTiter Non-Radioactive Cell Proliferation Assay was performed after 24 h, to determine effect of SM on cell growth. Growth rate was expressed as absorbance of the formazan product at 490 nm. Columns, mean; bars, SD; ^*^p<0.05; ^**^p<0.01.

Many epidemiological research studies have shown that cigarette smoking is linked to aggravation of cancer progression. Here, we examined the concentration of cigarette smoke medium which supported the growth of prostate cancer cells. Cigarette smoke medium (SM) was prepared as described in Materials and methods. Concentrations of total particulate materials (TPM), tar, nicotine (Nico) and carbon monoxide (CO) in SM were estimated from the Federal Trade Commission Smoking analysis as described in Materials and methods and indicated in Fig. 1. PC3 cells were plated onto 96-well plates containing 10% FBS culture medium. After 24 h, the cell culture medium was replaced with culture medium containing 0.5% FBS. After a further 24 h, cell cultures were treated with serial dilutions of SM ranging from 0.5–2% (v/v), for 48 h, as indicated in Fig. 1. Cell proliferation was assessed using the CellTiter Non-Radioactive Cell Proliferation Assay, known as MTS. Treatment with 0.5 and 1% SM significantly promoted growth of PC3 cells, however, 2% SM did not appear to have a significant inhibitory effect on cell proliferation (Fig. 1). SM (4, 10 and 20%) inhibited cell growth (data not shown). SM supported the growth of PC3 cells in a dose-dependent manner, in particular, SM (0.5%). SM (0.5%) in cell culture media was used for further studies.

### Cigarette smoke induced expression of HO-1 in prostate cancer cells

**Figure 2:**
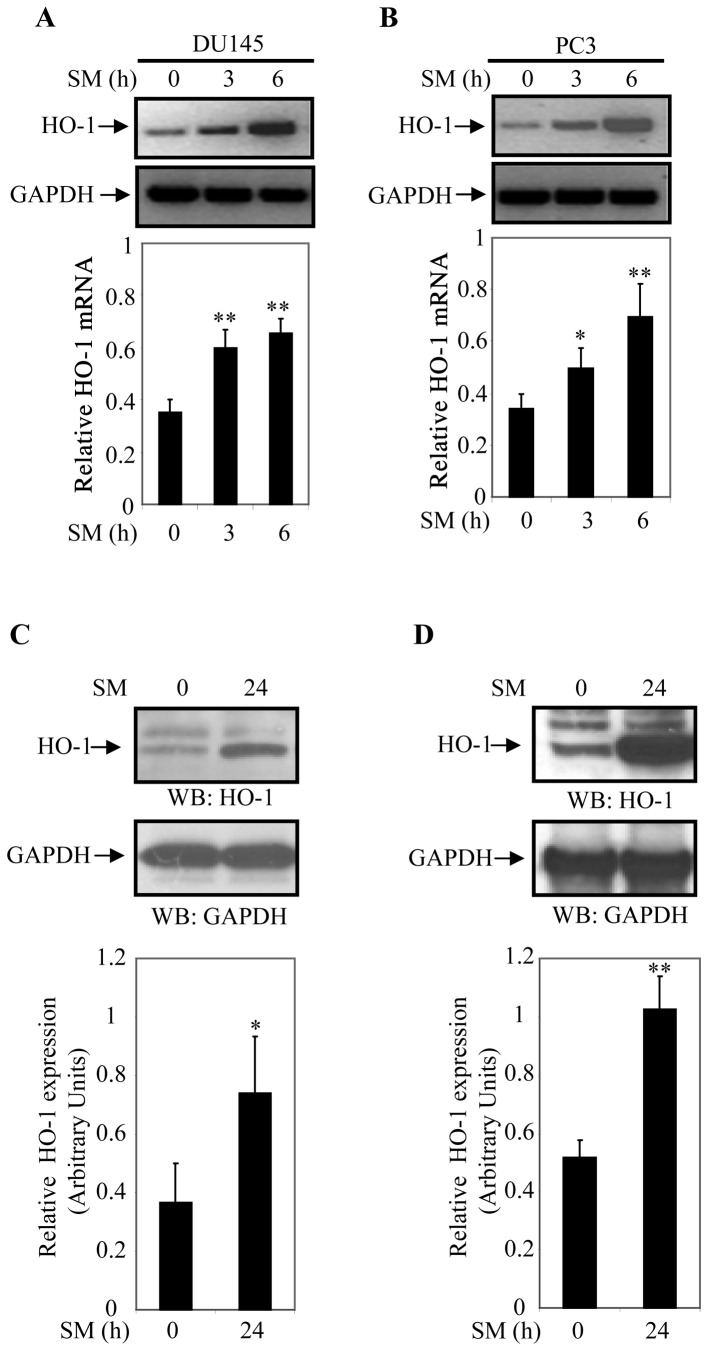
Cigarette smoke induced expression of HO-1 in prostate cancer cells. (A and B) SM induced mRNA levels of HO-1 in prostate cancer cells. DU145 and PC3 were treated with SM for 0, 3 and 6 h. Total RNA was isolated and analyzed by semi-quantitative RT-PCR using primers for HO-1 and GAPDH (internal control). Relative HO-1 mRNA levels derived from three individual experiments were expressed in arbitrary units. Columns, mean; bars, SD; ^*^p<0.05; ^**^p<0.01. (C and D) SM increased HO-1 protein levels in prostate cancer cells. (C) DU145 and (D) PC3 cells were grown on 6-well plates and treated with SM for 0 and 24 h. Cell extracts were subjected to western blot analysis using anti-HO-1 and anti-GAPDH antibodies. GAPDH served as an internal control. Relative HO-1 protein levels derived from three individual experiments were expressed in arbitrary units. Columns, mean; bars, SD; ^*^p<0.05; ^**^p<0.01.

Next, we examined whether SM induces expression of HO-1 in DU145 and PC3 cells. For this study, cells were treated for 0, 3 and 6 h with 0.5% SM. Steady state transcript levels of HO-1 and GAPDH (internal control) were assessed by semi-quantitative PCR analysis. HO-1 transcript levels increased in 0.5% SM-treated DU145 and PC3 cells in a time-dependent manner (Fig. 2A and B). HO-1 mRNA levels were 1.7- and 1.9-fold higher in DU145 and 1.5- and 2.0-fold higher in PC3 cells after 3 and 6 h of 0.5% SM treatment, respectively (Fig. 2A and B). These results suggested that HO-1 plays a central role in the response to SM exposure in prostate cancer cells.

To further analyze the effect of SM on activation of HO-1, DU145 and PC3 cells were treated with SM for 24 h and expression of HO-1 was determined by western blot analyses. Cell extracts of DU145 and PC3 cells treated with 0.5% SM for 0 and 24 h were prepared and protein expression was determined by western blot analysis using an anti-HO-1 antibody. Expression of HO-1 (normalized to GAPDH) was significantly higher in SM treated cells compared to controls (Fig. 2C and D). HO-1 expression was 2.1-fold (p=0.047) higher in DU145 cells and 2.0-fold (p=0.002) higher in PC3 cells after 24 h of 0.5% SM treatment compared to controls (0 h of treatment). Relative levels of HO-1 expression were determined based on data derived from three independent experiments (Fig. 2C and D). These finding indicated that 0.5% SM induced steady-state HO-1 mRNA and protein levels in DU145 and PC3 cells.

### Cigarette smoke induced VEGF secretion in prostate cancer cells

**Figure 3:**
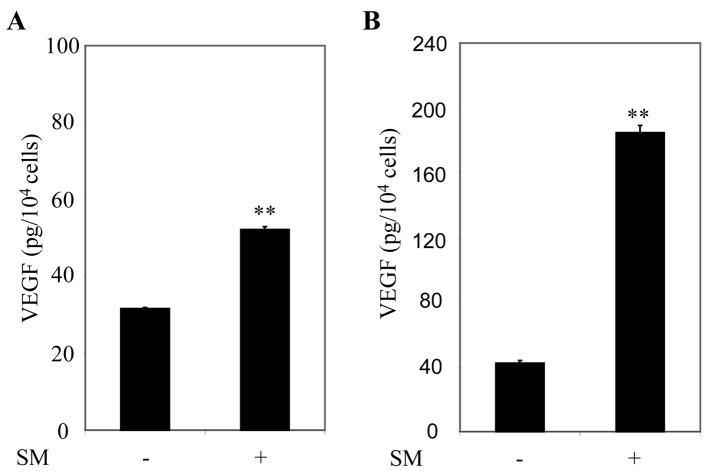
Cigarette smoke induced VEGF secretion in prostate cancer cells. (A) DU145 and (B) PC3 cells were grown in complete culture medium. Cells were refreshed with culture medium containing 0.5% FBS for 24 h, and then treated with SM. Culture supernatants were collected and cells were counted after 24 h. VEGF concentration was determined by ELISA and expressed as pg/10^4^ cells. Micrographs are representative of three individual experiments. Columns, mean; bars, SD; ^*^p<0.05; ^**^p<0.01.

VEGF has been implicated in tumor progression and expression of VEGF has been reported in a number of cell lines and clinical specimens derived from a broad range of cancers (30–33). Here we examined whether exposure to SM stimulated VEGF secretion in prostate cancer cells. DU145 and PC3 cells were grown in culture medium supplemented with 10% FBS. After 24 h, cells were refreshed with culture medium containing 0.5% FBS, and then treated with 0.5% SM for 0 and 24 h. Cell culture supernatants were collected after 0 and 24 h, and VEGF secretion was assessed by ELISA per the manufacturer’s instructions. VEGF increased 1.65-fold (p=0.0002) in DU145 cells (Fig. 3A) and 4.38-fold (p=0.0002) in PC3 cells (Fig. 3B) after 24 h treatment with SM. These results suggested that SM induced VEGF secretion in prostate cancer cells.

### Cigarette smoke induced nuclear translocation of HO-1 in prostate cancer cells

**Figure 4:**
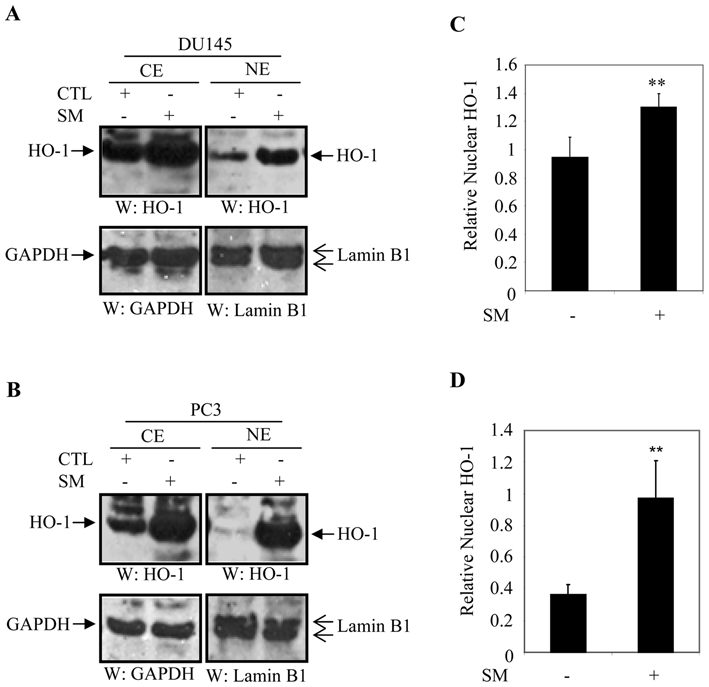
Cigarette smoke induced nuclear translocation of HO-1 in prostate cancer cells. (A) DU145 and (B) PC3 cells were grown on 6-well-plates and treated with SM. After 24 h, cellular fractionation was performed, and the cytoplasmic and nuclear fractions were analyzed by western blotting using an anti-HO-1 antibody. The blots were re-probed with anti-GAPDH or anti-Lamin B1 antibodies. (C) DU145 and (D) PC3 cells were treated with SM. Nuclear extracts were probed with anti-HO1 and Lamin B1 antibodies. Nuclear expression of HO-1 was normalized to that of the nuclear marker Lamin B1, and relative expression of nuclear HO-1 was expressed in arbitrary units. Data shown in micrographs were derived from three individual experiments. Columns, mean; bars, SD; ^**^p<0.01. CTL, control; CE, cytoplasmic extract; NE, nuclear extract.

Recent studies reported that nuclear localization of HO-1 was associated with prostate cancers (19), and head and neck squamous cell carcinomas (27). Treatment of DU145 and PC3 cells with SM induced mRNA and protein levels of HO-1 as compared to control counterparts (Fig. 2). We examined whether SM induced nuclear translocation of HO-1 in DU145 and PC3 cells. Cellular fractionation was performed to determine the cellular distribution of HO-1 in SM-treated and untreated cells. DU145 and PC3 cells were treated with either culture medium or 0.5% SM and cellular fractions were blotted with anti-HO-1, anti-GAPDH (cytoplasmic maker) or anti-lamin B1 (nuclear marker) antibody after 24 h. The level of cytoplasmic HO-1 was significantly higher in SM-treated DU145 and PC3 cells compared to their control counterparts (Fig. 4A and B).

We also observed that levels of HO-1 in the nuclear fraction significantly increased in DU145 and PC3 cells treated with SM compared to untreated controls (Fig. 4A and B). For quantification purposes, expression of nuclear HO-1 was normalized to that of lamin B1 and expressed in arbitrary units. Data were averaged from three independent experiments. Relative levels of nuclear HO-1 were 1.37-fold (p=0.001) higher in DU145 cells and 2.65-fold (p=0.01) higher in PC3 cells treated with 0.5% SM compared to controls (Fig. 4C and D).

### Nuclear-directed expression of HO-1 in HEK293 cells

**Figure 5:**
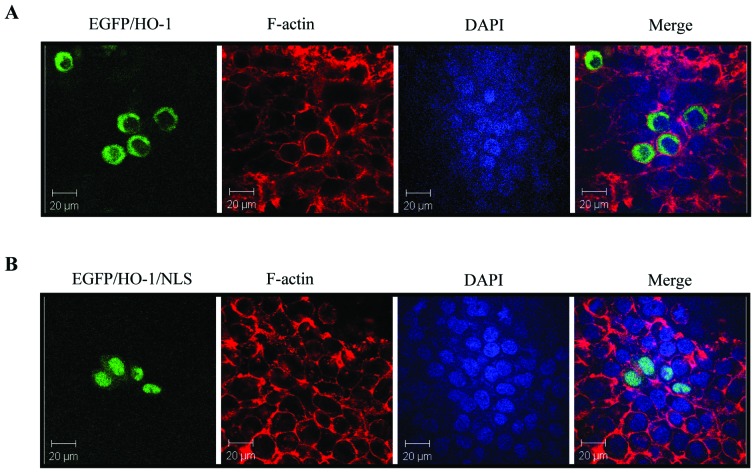
Nuclear-directed expression of HO-1 in HEK293 cells. HO-1 and HO-1/NLS were constructed into pEGFP-N3 vector. Resultant constructs, (A) pEGFP-HO-1 and (B) pEGFP-HO-1/NLS, were transfected in HEK293 cells. After 24 h, cells were fixed and stained with F-actin phalloidin and DAPI. Images were taken by using Zeiss LSM 510 Meta confocal microscope. Bar, 50 *μ*m.

SM increased cytoplasmic expression and nuclear translocation of HO-1 in prostate cancer cells (Fig. 4). SM-mediated expression of HO-1 also correlated with an increase in VEGF secretion (Fig. 3). These observations prompted us to investigate the role of cytoplasmic and nuclear HO-1 in the regulation of VEGF. For this study, we generated two constructs by fusing HO-1 and HO-1 with C-terminal nuclear localization signals (NLS) to the N-terminus of EGFP. The generated constructs were designated pEGFP-HO-1 and pEGFP-HO-1/NLS. HEK293 cells were transfected and stained with F-actin (cytoplasmic marker) and DAPI (nuclear marker). As anticipated, cells transfected with pEGFP-HO-1 expressed HO-1 exclusively in the cytosol (Fig. 5A), whereas cells transfected with pEGFP-HO-1/NLS expressed HO-1/NLS exclusively in the nucleus (Fig. 5B), demonstrating that a carboxyl terminal NLS could efficiently mediate nuclear expression of HO-1 *in vitro* (Fig. 5B). Exclusive expression of HO-1/NLS in the nucleus can therefore be used to examine the function of nuclear HO-1 *in vitro*, thus mimicking endogenous HO-1 in prostate cancer tissues as shown previously (19,33). Furthermore, this system can be used to compare function of cytoplasmic and nuclear HO-1 in VEGF transcriptional activation and secretion.

### Nuclear localization of HO-1 promoted transcriptional activity of VEGF

**Figure 6:**
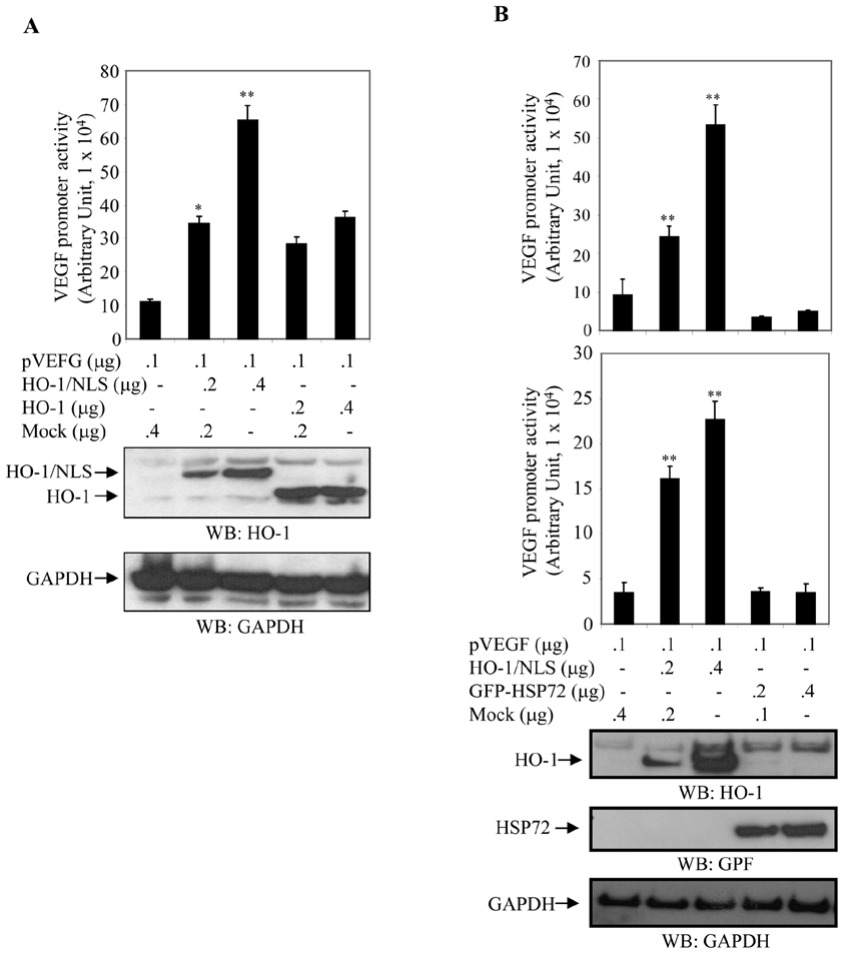
Nuclear localization of HO-1 promoted transcriptional activity of VEGF. (A) Differential activation of VEGF transcriptional activity by cytoplasmic and nuclear HO-1. HEK293 cells were co-transfected with the VEGF promoter and HO-1 or HO-1/NLS in a dose-dependent fashion, as indicated. After 24 h, VEGF promoter activity (luciferase activity) was measured and normalized to β-Gal activity. Relative VEGF promoter activity derived from three experiments was expressed in arbitrary units. Cell extracts were also blotted with anti-HO-1 and anti-GAPDH antibodies. Columns, mean; bars, SD; ^*^p<0.05; ^**^p<0.01. pVEGF, VEGF promoter; HO-1, plasmid expressing HO-1 in cytosol; HO-1/NLS, plasmid expressing nuclear HO-1; NLS, nuclear localization signal. (B) Differential activation of VEGF transcriptional activity by heat shock proteins. HEK293 and COS7 cells were co-transfected with the VEGF promoter and pNuc-HO-1/NLS or EGFP-HSP72 in a dose-dependent manner as indicated. After 24 h, luciferase activity was measured and normalized to β-galactosidase (β-Gal) activity to quantify VEGF promoter activity. Relative VEGF promoter activity was expressed in arbitrary units. Micrograph is representative of three independent experiments. Cell extracts were also blotted with anti-HO-1, anti-GFP and anti-GAPDH antibodies. GAPDH served as internal control. Columns, mean; bars, SD; ^*^p<0.05; ^**^p<0.01. HO-1, also known as heat shock protein 32; and HSP72, heat shock protein 72; NLS, nuclear localization signal. Upper panel: HEK293; lower panel: COS7.

Next, we generated two constructs of HO-1, pFlag-HO-1 (HO-1 with N-terminal FLAG-tag) and pNuc-HO-1/NLS (HO-1 with C-terminal NLS). We then assessed whether cytoplasmic or/and nuclear expression of HO-1 enhanced transcriptional activity of VEGF. HEK293 cells were co-transfected with the VEGF promoter (pVEGF) and either pFlag-HO-1 (cytoplasmic HO-1) or pNuc-HO-1/NLS (nuclear HO-1) in a dose-dependent manner (Fig. 6A). Cell extracts were prepared after 24 h, and luciferase activity normalized to β-Gal activity was assayed to measure VEGF promoter activity. The expression levels of HO-1 were determined by western blot analysis with anti-HO-1 antibody. Membranes were stripped and reblotted with an anti-GAPDH antibody to ensure equal loading. Relative VEGF promoter activity was approximately 6-fold higher in cells transfected with pNuc-HO-1 and approximately 3-fold higher in cells transfected with pFlag-HO-1 compared to their control counterparts (Fig. 6A).

HEK293 and COS7 cells were cotransfected with the VEGF promoter (pVEGF) and either pNuc-HO-1/NLS [HO-1, also known as Heat Shock Protein 32 (10)] or pEGFP-HSP72 (HSP72, heat shock protein 72) as a control in a dose-dependent manner. Cell extracts were prepared after 24 h and a luciferase assay was performed to measure VEGF promoter activity. Luciferase activity was normalized and expressed in arbitrary units relative to β-Gal activity. Expression levels of HO-1 and HSP72 were determined by western blot analysis with anti-HO-1 and anti-GFP antibodies. Blotting with anti-GAPDH antibody demonstrated equal loading of cell extracts. Relative luciferase activity was upregulated in a dose-dependent manner in cells transfected with HO-1/NLS, whereas luciferase activity was relatively unchanged in cells transfected with GFP-HSP72 (Fig. 6B). VEGF promoter activity was 2.60-fold (p<0.001) and 5.7-fold (p<0.001) higher in HEK293T cells transfected 0.2 and 0.4 *μ*g of pNuc-HO-1/NLS, respectively (Fig. 6B), and 4.61-fold (p<0.001) and 6.47-fold (p<0.001) higher when COS7 cells which were similarly cotransfected. In contrast, transfection of HEK293 and COS7 cells with GFP-HSP72 had no statistically significant effect on luciferase activity (Fig. 6B). These results showed that nuclear HO-1 significantly increased transcriptional activity of the VEGF promoter in a dose-dependent manner, while HSP72 had an insignificant effect on the activity of the VEGF promoter (Fig. 6B). These findings suggested that nuclear expression of HO-1 plays an important role in the transcriptional activity of VEGF.

### Ectopic expression of nuclear HO-1 promoted VEGF secretion in prostate cancer cells

**Figure 7:**
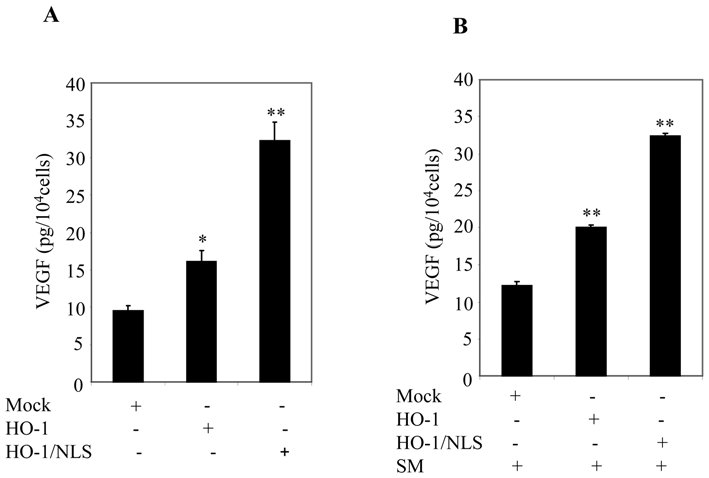
Ectopic expression of nuclear HO-1 promoted VEGF secretion in prostate cancer cells. (A) PC3 cells were transfected with mock, HO-1 or HO-1/NLS. After 24 h, cells were starved with cell culture medium containing 0.5% FBS. After 24 h, supernatants were collected, and VEGF secretions were measured by using ELISA. Columns, mean; bars, SD; ^*^p<0.05; ^**^p<0.01. (B) Cell culture, in (A), then were replenished with cell culture medium containing 0.5% FBS plus SM. After 24 h, supernatants were collected and VEGF secretions were measured by using ELISA. Columns, mean; bars, SD; ^*^p<0.05; ^**^p<0.01.

Given that ectopic expression of nuclear HO-1 increased VEGF promoter activity (Fig. 6), we sought to further examine the effect of nuclear HO-1 on VEGF secretion using ELISA. For this analysis, PC3 cells were used because of their high transfection efficiency. PC3 cells were transfected with mock, pFlag-HO-1 or pNuc-HO-1/NLS. Cell cultures were washed and replaced with fresh medium containing 0.5% FBS within 24 h. Cell culture supernatants were collected and analyzed for VEGF using ELISA. Ectopic expression of HO-1/NLS significantly enhanced VEGF secretion compared to cells transfected with HO-1 (Fig. 7A). Next, cell cultures were replaced with fresh medium containing 0.5% FBS and then treated with SM for 24 h. Supernatants were collected and VEGF concentration was measured by ELISA. A significant increase in VEGF secretion was observed in cells transfected with HO-1/NLS compared to cells transfected with mock or HO-1 (Fig. 7B). These data suggested that nuclear HO-1 was involved in promoting VEGF secretion.

## Discussion

Cigarette smoking represents one of the most serious problems for public health, and at present accounts for 6 million deaths annually worldwide (34). Although the relevance of this epidemic is known, the molecular mechanism(s) underlying its toxicity and carcinogenic potential remain elusive. Cigarette smoking has been linked to cancers of the lung, breast and brain (34), but while some research studies have shown that cigarette smoking is not associated with the incidence of prostate cancer, other reports suggested that current or recent cigarette smoking is linked to an elevated risk of mortality, advanced stage or high-grade disease (6). Cigarette smoking may therefore be involved in the progression rather than the initiation of prostate cancer. A number of studies demonstrated that angiogenesis is associated with the progression of prostate cancer (31,32). Previous reports detected higher levels of HO-1 protein in various tumor tissues compared to normal tissue (33,35–40), and was associated with tumor progression of head and neck squamous cell carcinomas (27). The angiogenic cytokine VEGF has a central role in tumor angiogenesis by binding and activating the receptors, VEGFR1 and VEGFR2. The VEGF/VEGFR axis promotes endothelial cell differentiation, cell growth, tubular formation and migration (41–45). It has also been reported that HO-1 is an important proangiogenic mediator, which further supports tumor progression (12). The present study explores the relationship between cigarette smoke, HO-1 expression and VEGF secretion in prostate cancer cells.

In response to oxidative stress, cells have evolved multiple protective mechanisms to neutralize and clear toxic molecules and restore cellular redox homeostasis. Induction of HO-1 is a fundamental cellular defense process against oxidative stress caused by environmental stimuli. Cells lacking HO-1 are susceptible to free radical damage and oxidative injury, which results in high levels of endothelial damage and prolonged inflammation (10,11). Treatment with SM induced HO-1 mRNA expression and increased HO-1 protein in prostate cancer cells (Fig. 2). Induction of HO-1 may therefore provide the first line of cellular defense of prostate cancer cells against the oxidative stimulus of cigarette smoke. Thus, an increase in HO-1 levels may be required for survival of prostate cancer cells. This result is consistent with previous analyses of HO-1 expression in different types of cancer. Overexpression of HO-1 has been reported in lymphosarcoma (35), brain tumors (36), renal carcinoma (37), hepatoma (38), Kaposi sarcoma (39), pancreatic cancer (40) and chronic myeloid leukemia (41). Cigarette smoking has been shown to be associated with an elevated risk of mortality or advanced stage prostate cancer, but not with incidence of prostate cancer (6). These lines of evidence strongly suggest that SM-mediated induction of HO-1 may be associated with the progression of prostate cancer.

HO-1 has been reported to promote VEGF secretion and facilitate VEGF-mediated activities such as promoting the efficiency of cell proliferation and migration and improving the formation of capillary-like tubular structures and capillary outgrowth (14,15). Treatment of prostate cancer cells with SM induced expression of HO-1 (Fig. 2) and enhanced VEGF secretion in both DU145 and PC3 cells (Fig. 3). Ectopic expression of HO-1 (also known as heat shock protein 32) and another heat shock protein, HSP72 were tested for their ability to induce VEGF transcriptional activity. While HSP72 failed to increase transcriptional activity of VEGF, HO-1 induced significant transcriptional activity of VEGF (Fig. 6) and VEGF secretion (Fig. 7). These data suggested that cigarette smoke may therefore promote the progression of prostate cancer through HO-1-modulated VEGF increase.

Previous studies reported that nuclear translocation of HO-1 was associated with prostate cancer (19), and its nuclear expression had a strong correlation with the grade of differentiation of oral squamous cell carcinomas (28,29) and the tumor progression of head and neck squamous cell carcinomas (27). Together with our finding that SM induced nuclear translocation in prostate cancer, this suggested that SM-mediated nuclear localization of HO-1 is contributable to the progression of prostate cancer. Given that nuclear localization may be associated with the progression of prostate cancer, we asked whether nuclear HO-1 was involved in the promotion of VEGF secretion in prostate cancer. To address this question we attempted to express HO-1 exclusively in nuclei of cells by tagging it with tandem nuclear localization signals. Interestingly, ectopic expression of nuclear HO-1 had the potential to significantly induce VEGF transcriptional activity and secretion, whereas cytoplasmic HO-1 did not (Fig. 6). Taken together, these findings strongly suggest that nuclear localization of HO-1 induced by cigarette smoke plays a central role in VEGF secretion and may contribute to tumor angiogenesis and the progression of prostate cancer.

Our study revealed the mechanism by which cigarette smoking is associated with prostate cancer through nuclear HO-1 and VEGF regulation. This study also provides new insights into the involvement of cigarette smoke and HO-1 in prostate cancer. Therapies targeting nuclear HO-1 may therefore represent a novel approach for the treatment of prostate cancer.
